# Butyrate and iso-butyrate: a new perspective on nutrition prevention of gestational diabetes mellitus

**DOI:** 10.1038/s41387-024-00276-4

**Published:** 2024-04-25

**Authors:** Weiling Han, Jia Wang, Xin Yan, Cheng Liu, Junhua Huang, Lirui Zhang, Yujie Zhang, Yiqing Zhao, Yanmei Hou, Wei Zheng, Guanghui Li

**Affiliations:** 1 Department of Obstetrics, Beijing Obstetrics and Gynecology Hospital, Capital Medical University, Beijing, China; 2 Beijing Maternal and Child Health Care Hospital, Beijing, China; 3 Hyproca Nutrition Co., Ltd, Changsha, Hunan China

**Keywords:** Gestational diabetes, Risk factors

## Abstract

**Background:**

Dietary imbalance, such as a lower proportion of complex carbohydrates and a higher protein diet, may contribute to gestational diabetes mellitus (GDM) risks through their metabolisms. However, there is a lack of knowledge regarding the association between butyrate, iso-butyrate, and GDM, which are metabolisms of the two primary nutrients above. This study aimed to clarify the association of butyrate and iso-butyrate with GDM.

**Methods:**

A nested case–control study was conducted based on the Beijing Birth Cohort Study (BBCS) from 2017 to 2018. Totally, 99 singleton women were involved (GDM: *n* = 49, control: *n* = 50). All participants provided blood samples twice (in their first and second trimesters). Gas chromatography-mass spectrometry (GC-MS) was used for butyrate and iso-butyrate detection. Unconditional logistic regression and receiver operating characteristic (ROC) curve analysis were used for statistical analysis.

**Results:**

The results showed that butyrate in the first trimester was negatively correlated with GDM (odds ratio (OR): 0.00, 95% confidential interval (CI): 0.00–0.21, *P* = 0.008), and iso-butyrate in the second trimester was positively related to GDM (OR: 627.68, 95% CI: 40.51–9724.56, *P* < 0.001). The ratio (butyrate/iso-butyrate) was negatively associated with GDM, both in the first trimester (OR: 0.00, 95%CI: 0.00–0.05, *P* < 0.001) and in the second trimester (OR: 0.52, 95% CI: 0.34–0.80, *P* = 0.003). The area under the curve (AUC) using the ratio in the first trimester combined with clinical risk factors achieved 0.89 (95% CI: 0.83–0.95). Iso-butyrate in the second trimester combined with clinical risk factors achieved an AUC of 0.97 (95% CI: 0.92–1.00).

**Conclusions:**

High iso-butyrate and low butyrate levels may be associated with an increased risk of GDM. As they are produced through dietary nutrient formation by gut microbiota, further studies on the association of dietary intake and butyrate or iso-butyrate concentration in plasma may help find a novel approach to nutritional intervention for GDM.

## Introduction

Gestational diabetes mellitus (GDM), one of the most common gestational complications, affects approximately 14% of pregnancies around the world [1]. The prevalence of GDM in mainland China was 14.8% and varied across different cities and regions of China [2]. After the “two-child policy” and “three-child policy” were put into effect in China, pregnant women with advanced age, GDM history, or obesity increased, resulting in higher and higher morbidity of GDM [3, 4]. Besides higher risks of adverse perinatal outcomes, GDM also leads to higher susceptibility to metabolic diseases for mothers and children in the long run [5]. Early detection and timely intervention are of great clinical significance in improving GDM outcomes [6]. The association of metabolites with GDM and their potential profitable effects on GDM have drawn great attention [7, 8].

Dietary factors, specifically non-digestible carbohydrates and dietary protein, have been linked to the risks of GDM [9]. Butyrate, metabolites of non-digestible carbohydrates, and iso-butyrate, metabolites derived from the fermentation of dietary protein (mainly branched-chain amino acids, BCAAs) [10, 11], have been demonstrated to have crucial roles in regulating glucose homeostasis [12, 13]. Despite the similar ingredients of butyrate and iso-butyrate, both containing four carbons, variations in fermentation substrates can result in contrasting effects on metabolic regulation [14–16]. Extensive research has unveiled the advantageous effects of butyrate on glucose metabolism in non-pregnant patients with type 2 diabetes [17, 18], while increased proteolytic fermentation, (products such as iso-butyrate, isovaleric acid, phenols, indoles, and amines) is thought to be mainly associated with harmful health effects [19–21]. However, the association of butyrate and iso-butyrate with GDM remains inconsistent [22–24].

This study aimed to investigate the prospective association of butyrate and iso-butyrate with GDM, and explore the incremental predictivity of the two nutrients-related metabolites for GDM risk beyond conventional risk factors.

## Materials/subjects and methods

### Study design and population

**Fig. 1:**
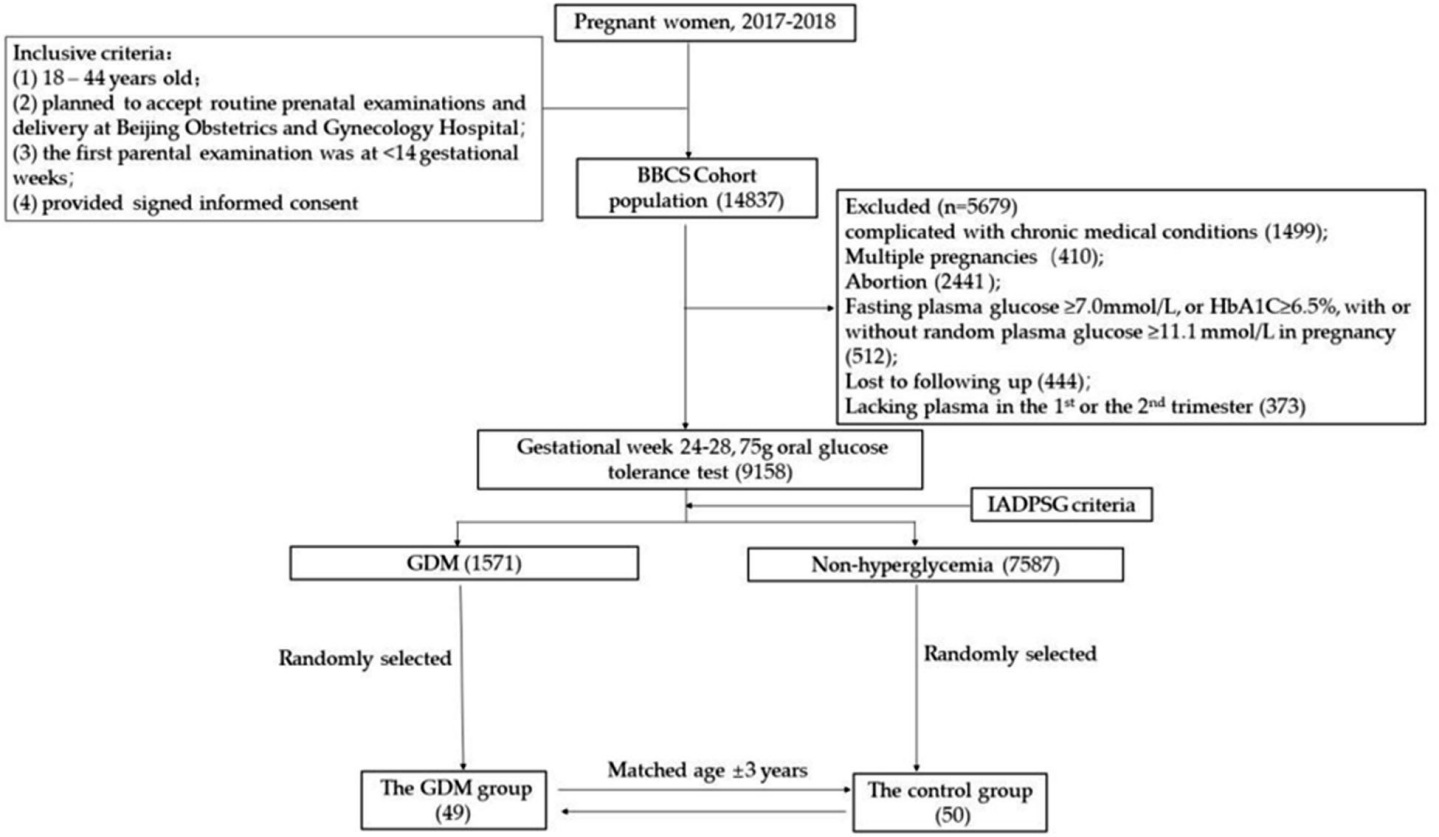
Flowchart of participants. GDM gestational diabetes mellitus, BBCS cohort the Beijing Birth Cohort Study; chronic medical conditions including hypertension, pre-pregnancy diabetes, heart disease, liver disease, kidney diseases, thyroid disease, or mental illness. GDM was diagnosed when any of the following criteria were met: fasting plasma glucose ≥ 5.1 mmol/L, plasma glucose at 1 h ≥ 10.0 mmol/L, or plasma glucose at 2 h ≥ 8.5 mmol/L, according to the International Association of Diabetes and Pregnancy Study Groups (IADPSG) criteria.

A nested case–control study was conducted based on a prospective maternal and child health cohort, the Beijing Birth Cohort Study (BBCS, ChiCTR2200058395), from 2017 to 2018 in Beijing, China. The inclusive criteria have been described in a previous study [25]. Briefly, pregnant women were recruited if they met the following criteria: aged 18–44, with gestational week <14 weeks at their first visit for prenatal examination, with a written informed consent, and women who planned to accept routine prenatal examinations and delivery at Beijing Obstetrics and Gynecology Hospital. Women were excluded if they met the following criteria: complicated with chronic medical conditions (including hypertension, diabetes, thyroid disease, heart disease, liver and kidney diseases) or mental illness; abortion; multiple pregnancies; fasting plasma glucose (FPG) ≥ 7.0 mmol/L, or HbA1C ≥ 6.5%, with or without random plasma glucose ≥11.1 mmol/L in pregnancy; lost to following-up; without blood samples in the first or second trimester. All participants provided fasting blood samples twice, in gestational week <14 weeks (T1, the first trimester) and gestational 14–27^+6^ weeks (T2, the second trimester) respectively. A 75 g oral glucose tolerance test (OGTT) was performed at gestation 24–28 weeks and GDM was diagnosed when any of the following criteria were met: FPG ≥ 5.1 mmol/L, plasma glucose at 1 h ≥ 10.0 mmol/L, or plasma glucose at 2 h ≥ 8.5 mmol/L, according to the International Association of Diabetes and Pregnancy Study Groups (IADPSG) criteria [26]. Subjects were randomly chosen in the GDM and control group (1:1) with age (±3) matched. A total of 99 participants (49 in the GDM group and 50 in the control group) were involved in the final analysis (Fig. 1).

The study was approved by the Ethics Committee of Beijing Obstetrics and Gynecology Hospital, Capital Medical University (2022-KY-088-01). All procedures were conducted according to the guidelines in the Declaration of Helsinki.

### Data collection and definitions

Pre-pregnancy body mass index (BMI) was calculated based on self-reported height and pre-pregnancy weight. BMI was classified into three grades as follows: slim: BMI < 18.5 kg/m^2^; normal: 18.5 kg/m^2^ ≤ BMI < 24 kg/m^2^; overweight: BMI ≥ 24 kg/m^2^ [27]. Smoking history was defined as at least one cigarette a day for at least six months. Drinking history referred to drinking alcohol at least once a month for six months or more. Gestational weight gain until 28 weeks was obtained through weight at gestational 28 weeks minus pre-pregnancy weight. A family history of diabetes was defined as a first-degree relative with diabetes. History of illness mainly refers to high-risk factors related to GDM, including GDM in a previous pregnancy, stillbirth or fetal death, birth defect, macrosomia, polyhydramnios, spontaneous abortion, polycystic ovarian syndrome (PCOS), and vulvovaginal candidiasis. Stillbirth or fetal death referred to fetal death at 20 weeks or greater of gestation [28]. Birth defect was defined as any structural or functional abnormalities during embryonic or fetal development [29]. Macrosomia was characterized as a birth weight equal to or greater than 4000 × *g* [30]. Polyhydramnios was diagnosed as amniotic fluid index ≥25 cm [29]. Spontaneous abortion was defined as the loss of an intrauterine pregnancy before viability [31]. The revised Rotterdam diagnostic criteria were used to diagnose PCOS [32]. Vulvovaginal candidiasis was diagnosed if 5 or more properties were met with the following criteria: soreness, dyspareunia, positive vaginal swab either at presentation or in the past, previous response to antifungal medication, exacerbation with antibiotics, cyclicity, swelling, and discharge [33]. All of the clinical information mentioned above were collected at the first time they went to hospital for prenatal examination and were recorded in the electronic clinical system. The FPG and lipid profiles, including cholesterol (CHOL), triglyceride (TG), high-density lipoprotein (HDL), and low-density lipoprotein (LDL), were determined as described in a previous study [34].

### Butyrate and iso-butyrate analysis

All fasting blood samples were centrifuged at 1680 × *g* for 10 min within 2 h after collection, and serum samples were extracted and stored at −80 °C for further examination. Gas chromatography-mass spectrometry (GC-MS) was used here for butyrate and iso-butyrate detection, and an ISQ 7610 single quadrupole GC-MS system (Thermo Fisher Scientific, USA) was employed for subsequent GC-MS analysis. A serum sample of 100 μl mixed with 10 μl of 10% sulfuric acid was placed into a 0.5 ml glass centrifuge tube for acidification. After oscillating for 30 s and leaving for 5 min, 100 μl of an ether mixed with isoamyl alcohol (internal standard, purity >98%, sigma, USA) (45.9 μmol/L) was added and shaken sufficiently. Then, centrifugation was performed at 19,064 × *g* for 10 min at 4 °C (MIKRO 220 R, Hettich, Germany). The upper layer of the ether phase was extracted. Butyrate and iso-butyrate were separated on an FFAP elastic quartz capillary column (30 m × 0.25 mm × 0.25 μm) from interfering substances in the matrix. The temperature program was set to rise from 60 °C to 180 °C at a rising rate of 15 °C per minute and retained for 2 min. High-purity nitrogen (purity 99.999%) was used as the carrier gas at a flow rate of 0.89 ml/min. The inlet temperature was at 200 °C. 1 μl of samples or butyrate and iso-butyrate standard (purity > 98%, sigma, USA) was injected in split injection mode at a ratio of 15:1. Standard curve was established and analyzed with series gradients of butyrate or iso-butyrate standards (1078 μmol/l, 539 μmol/l, 107.8 μmol/l, 53.9 μmol/l, 10.78 μmol/l). The mass spectrometry conditions were set as follows: ion source: electron ionization (EI); electron energy: −70 eV; ion source temperature: 230 °C; interface temperature: 250 °C; voltage of electron multiplier: 0.95 kV; solvent delay time: 3 min. Data were collected in scan mode with a range of 30–200 m/z.

### Sample size estimation

According to a previous study and our pre-experiment results, the ratio of butyrate to iso-butyrate was 1.62 ± 0.48 in the first trimester in pregnant women without complications [35]. The ratio decreased by 20% in the GDM group was considered a significant difference. Assuming that α = 0.05, β = 0.2, two-tailed test, the sample size was at least 35 in each group, calculated by the PASS 2021 software (PASS 2021 Power Analysis and Sample Size Software (2021)). A total of 99 participants (49 in the GDM group and 50 in the control group) were involved in our study, which was sufficient for the final analysis.

$$n=\frac{{({{\rm{Z}}}_{\alpha }+{{\rm{Z}}}_{\beta })}^{2}* 2{\sigma }^{2}}{{{\rm{\delta }}}^{2}}$$

### Statistical analysis

All participants were singleton pregnant women, and sex was not considered a factor in the statistical analysis of the data. Mean with standard deviation (SD) for normal distribution and median with interquartile range (IQR) for skewed distribution were used for statistical description. Categorical variables were described as numbers and proportions. Student’s *t* test, the Mann–Whitney U test, or Pearson’s Chi-square test were used to compare the difference between GDM and the control group. Wilcoxon’s sign-Rank test was used for concentration comparison between T1 and T2 in the longitudinal term. Unconditional logistic regression was used to analyze the association of butyrate, iso-butyrate, and butyrate/iso-butyrate with GDM with or without adjustment for clinical confounders (age, pre-pregnancy BMI, FPG, CHOL, TG, and LDL in T1). Clinical confounders that may affect the risk of GDM were screened out by univariate logistic regression (Supplementary Table S1). The odds ratio (OR) and 95% confidence interval (CI) were used to quantify the risk of GDM. The receiver operating characteristic (ROC) curve analysis was performed to clarify the predictive potential for GDM. The area under the curve (AUC) was calculated to evaluate the accuracy, sensitivity and specificity of the model. SPSS 26.0 (Released 2019. IBM SPSS Statistics for Windows, Version 26.0, IBM Corp, Armonk, New York, NY, USA) was used for data analysis, and a two-tailed *p*-value < 0.05 was considered statistically significant.

## Results

### Baseline information

The incidence of GDM in the cohort was 17.2% (1571/9158). A total of 99 singleton women were involved in the final analysis (*n* = 49 in the GDM group, *n* = 50 in the control group). Pre-pregnancy BMI was significantly higher in the GDM group (22.38 vs. 20.79 kg/m^2^, *P* = 0.003), with a larger proportion of women with overweight (32.7% vs. 6.0%). FPG, CHOL, TG, and LDL in T1 were significantly higher in the GDM group (FPG: 4.86 vs. 4.65 mmol/L, *P* = 0.020; CHOL: 4.46 vs. 4.11 mmol/L, *P* = 0.012; TG: 1.36 vs. 1.10 mmol/L, *P* = 0.015; LDL: 2.34 vs. 2.06 mmol/L, *P* = 0.014). There was no significant difference between the two groups in age, education, gravida, parity, smoking, drinking, family history of diabetes, history of illness, gestational weight gain (GWG) until 28 weeks, and sampling time (around 8 weeks in T1 and 24 weeks in T2) (*P* > 0.05) (Table 1). All women were not antibiotic-treated during pregnancy.

**Table 1:** Baseline characteristics of participants.

Characteristics	Total (*N* = 99)	GDM (*N* = 49)	Control (*N* = 50)	*P* value
	*n* (%)	*n* (%)	*n* (%)	
Age (year)^a^	32.68 ± 3.57	33.37 ± 3.97	32.00 ± 3.02	0.056
Pre-pregnancy BMI (kg/m^2^)^a^	21.58 ± 2.72	22.38 ± 2.71	20.79 ± 2.52	0.003
Slim	14 (14.1)	3 (6.1)	11 (22.0)	0.001
Normal	66 (66.7)	30 (61.2)	36 (72.0)	
Overweight	19 (19.2)	16 (32.7)	3 (6.0)	
Education				
Master’s or higher	29 (29.3)	15 (30.6)	14 (28.0)	0.951
Bachelor’s	52 (52.5)	25 (51.0)	27 (54.0)	
Lower than bachelor’s	18 (18.2)	9 (18.4)	9 (18.0)	
Economy				
>20,000	36 (37.1)	13 (27.7)	23 (46.0)	0.040
10,000–19,999	41 (42.3)	26 (55.3)	15 (30.0)	
<10,000	20 (20.6)	8 (17.0)	12 (24.0)	
Gravida				
1	46 (46.5)	20 (40.8)	26 (52.0)	0.375
2	32 (32.3)	16 (32.7)	16 (32.0)	
≥3	21 (21.2)	13 (26.5)	8 (16.0)	
Parity				
Nulliparous	66 (66.7)	32 (65.3)	34 (68.0)	0.776
Multiparous	33 (33.3)	17 (34.7)	16 (32.0)	
Smoking				
Yes	3 (3.1)	2 (4.3)	1 (2.0)	0.941
No	93 (96.9)	44 (95.7)	49 (98.0)	
Drinking				
Yes	9 (9.4)	5 (10.9)	4 (8.0)	0.895
No	87 (90.6)	41 (89.1)	46 (92.0)	
Family history of diabetes				
Yes	17 (17.2)	11 (22.4)	6 (12.0)	0.168
No	82 (82.8)	38 (77.6)	44 (88.0)	
History of illness^c^				
Yes	17 (17.2)	11 (22.4)	6 (12.0)	0.168
No	82 (82.8)	38 (77.6)	44 (88.0)	
GWG (until 28 weeks)^a^	8.32 ± 3.23	8.63 ± 3.50	8.04 ± 2.97	0.374
Sampling time				
First-trimester^b^	8 (3)	8 (2)	8 (2)	0.395
Second-trimester^b^	24 (1)	24 (1)	24 (1)	0.650
Laboratory parameters in the first trimester				
FPG (mmol/L)^a^	4.75 ± 0.44	4.86 ± 0.49	4.65 ± 0.37	0.020
CHOL (mmol/L)^a^	4.28 ± 0.71	4.46 ± 0.73	4.11 ± 0.65	0.012
TG (mmol/L)^a^	1.23 ± 0.54	1.36 ± 0.61	1.10 ± 0.41	0.015
HDL (mmol/L)^a^	1.48 ± 0.29	1.48 ± 0.30	1.48 ± 0.30	0.962
LDL (mmol/L)^a^	2.20 ± 0.56	2.34 ± 0.60	2.06 ± 0.49	0.014

*P* value < 0.05 for statistical significance.

*GDM* gestational diabetes mellitus, *BMI* body mass index; slim: BMI < 18.5 kg/m^2^; normal: 18.5 ≤ BMI < 23.9 kg/m^2^; overweight: BMI ≥ 24 kg/m^2^, *GWG* gestational weight gain, *FPG* fasting plasma glucose, *CHOL* cholesterol, *TG* triglyceride, *HDL* high-density lipoprotein, *LDL* low-density lipoprotein.

^a^Mean and standard deviation for descriptive statistics.

^b^Median and interquartile range for descriptive statistics.

^c^History of illness mainly referred to high risk factors related with GDM, including GDM in previous pregnancy, still birth, fetal death, birth defect, macrosomia, polyhydramnios, spontaneous abortion, polycystic ovarian syndrome, and vulvovaginal candidiasis.

### Comparison of butyrate and iso-butyrate in the GDM and control group

**Fig. 2:**
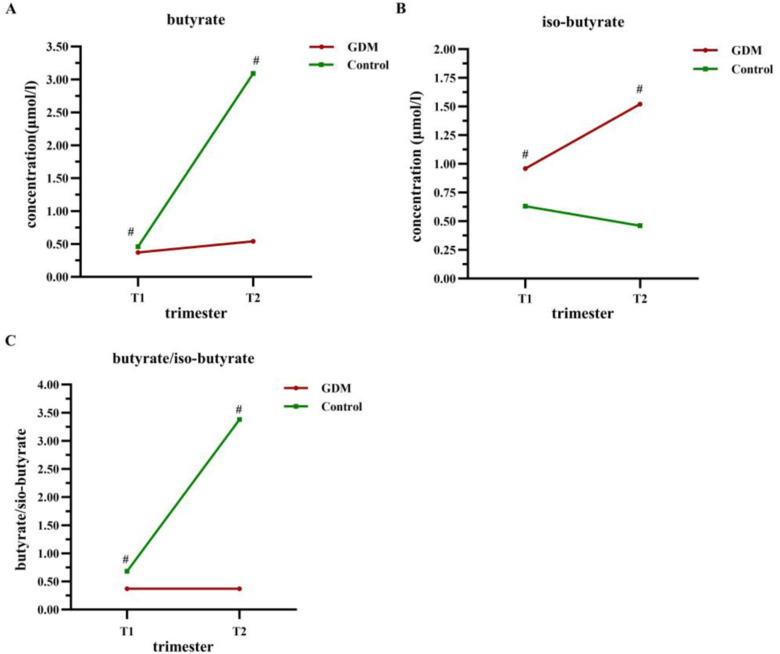
Dynamic Changes of butyrate, iso-butyrate, and butyrate/iso-butyrate from the first trimester to the second trimester. The tendency of butyrate (**A**), iso-butyrate (**B**), and butyrate/iso-butyrate (**C**) from the first trimester (T1) to the second trimester (T2). Lines of red representing the GDM group and lines of green representing the control group; GDM gestational diabetes mellitus. T1 the first trimester, T2 the second trimester; ^#^*P* ≤ 0.001.

The concentration of butyrate was significantly lower in the GDM group than that in the control group, both in T1 (0.37 vs. 0.46 μmol/L, *P* < 0.001) and T2 (0.54 vs. 3.09 μmol/L, *P* = 0.001) (Table 2). Though butyrate levels increased in both groups, it was much more apparent in the control group (Fig. 2A). The iso-butyrate level was higher in the GDM group in T1 (0.96 vs. 0.63 μmol/L, *P* < 0.001), and the difference was much more apparent (1.52 vs. 0.46 μmol/L, *P* < 0.001) in T2 (Table 2). Moreover, a reversed tendency of iso-butyrate from T1 to T2 was found in the two groups, as it was increased in the GDM group and decreased in the control group (Fig. 2B). Besides, there was a significantly increase of butyrate/iso-butyrate from T1 to T2 in the control group, while no apparent change was found in the GDM group (Fig. 2C).

**Table 2:** Association of butyrate, iso-butyrate, and butyrate/iso-butyrate with GDM.

Parameters	Total	GDM	control	*P* value^a^	Crude OR^b^	*P* value	Adjusted OR^c^	*P* value
	Median (IQR)	Median (IQR)	Median (IQR)		(95% CI)		(95% CI)	
Butyrate (μmol/L)								
First Trimester	0.40 (0.14)	0.37 (0.12)	0.46 (0.16)	<0.001	0.00 (0.00–0.03)^d^	<0.001	0.00 (0.00–0.21)^e^	0.008
Second Trimester	1.48 (3.50)	0.54 (2.90)	3.09 (3.98)	0.001	0.77 (0.62–0.96)	0.020	0.81 (0.64–1.04)	0.096
*P* value^e^	<0.001	<0.001	<0.001					
Iso-butyrate (μmol/L)								
First Trimester	0.94 (0.41)	0.96 (0.09)	0.63 (0.44)	<0.001	1.78 (0.64–4.93)	0.270	2.04 (0.78–5.34)	0.148
Second Trimester	1.39 (1.06)	1.52 (0.12)	0.46 (0.23)	<0.001	446.62 (50.83–3924.03)	<0.001	627.68 (40.51–9724.56)	<0.001
*P* value^e^	<0.001	<0.001	0.007					
Butyrate/Iso-butyrate								
First Trimester	0.45 (0.36)	0.37 (0.11)	0.68 (0.44)	<0.001	0.00 (0.00–0.03)^d^	<0.001	0.00 (0.00–0.05)^e^	<0.001
Second Trimester	1.68 (3.58)	0.37 (1.80)	3.38 (7.59)	<0.001	0.53 (0.37–0.75)	<0.001	0.52 (0.34–0.80)	0.003
*P* value^e^	<0.001	0.018	<0.001					

*P* value < 0.05 for statistical difference.

*GDM* gestational diabetes mellitus, *IQR* interquartile range, *OR* odds ratio, *CI* confidence interval.

^a^Mann–Whitney U test between GDM and control group.

^b^Single factor unconditional logistic regression.

^c^Unconditional logistic regression with adjustments for age, pre-pregnancy body mass index, cholesterol, triglyceride, low-density lipoprotein, and fasting plasma glucose in the first trimester.

^d^0.00 referred that the OR ≤ 0.01.

^e^Wilcoxon signed rank test between the first and second trimester in GDM and control population.

### Association of butyrate, iso-butyrate, and butyrate/iso-butyrate with the risk of GDM

Butyrate may significantly reduce the risk of GDM (T1, OR: 0.00, 95% CI: 0.00–0.03, *P* < 0.001; T2, OR: 0.77, 95% CI: 0.62–0.96, *P* = 0.020). Iso-butyrate in T2 can increase GDM risk to 446.62 (95% CI: 50.83–3924.03, *P* < 0.001). Butyrate/iso-butyrate was also negatively correlated with the risk of GDM, both in T1 (OR: 0.00, 95% CI: 0.00–0.03, *P* < 0.001) and T2 (OR: 0.53, 95% CI: 0.37–0.75, *P* < 0.001). After adjustment for clinical confounders, butyrate/iso-butyrate was still negatively correlated with GDM, Both in T1 (OR: 0.00, 95% CI: 0.00–0.05, *P* < 0.001) and in T2(OR: 0.52, 95% CI: 0.34–0.80, *P* = 0.003) (Table 2).

### The prediction effect of butyrate, iso-butyrate, and butyrate/iso-butyrate for GDM

**Fig. 3:**
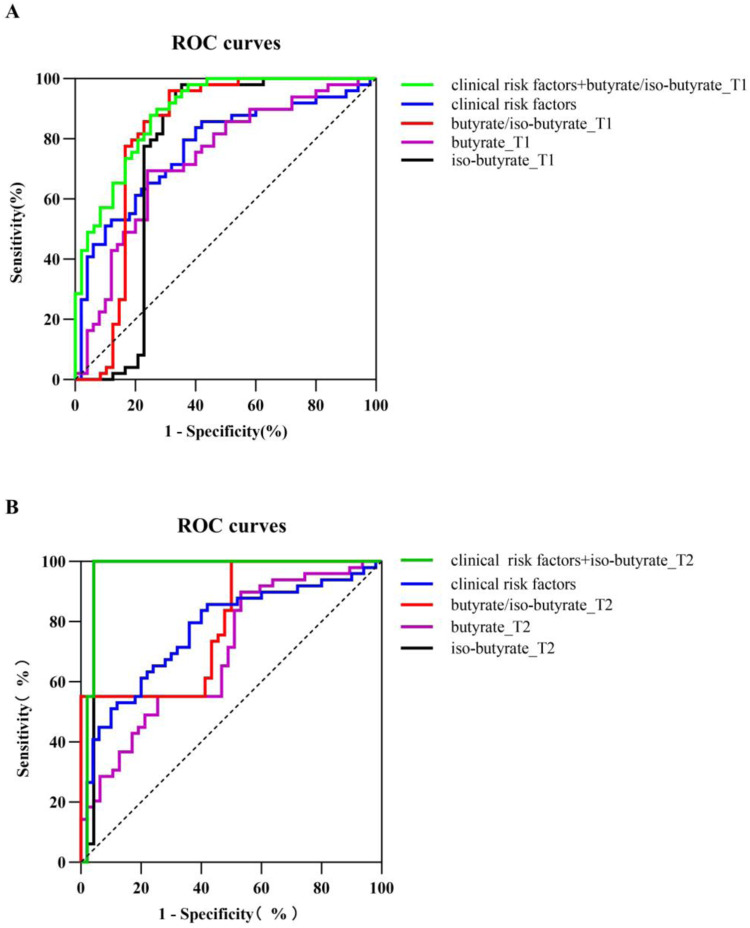
ROC curves for early prediction of GDM. Receiver operating characteristic (ROC) curves for logistic regression models in the first trimester (**A**) and in the second trimester (**B**). T1 the first trimester; T2 the second trimester; predictive models were built using clinical risk factors (lines of blue), butyrate/iso-butyrate (lines of red), butyrate (line of purple), iso-butyrate (line of black), clinical risk factors combined with butyrate/iso-butyrate in T1 or iso-butyrate in T2 (line of black); clinical risk factors included age, pre-pregnancy body mass index, cholesterol, triglyceride, low-density lipoprotein, and fasting plasma glucose in the first trimester.

Compared with butyrate alone or iso-butyrate alone for GDM prediction, ROC curves using butyrate/iso-butyrate in T1 showed a better predictive power with an AUC of 0.81 (95% CI: 0.71–0.91; butyrate, AUC: 0.73, 95% CI: 0.63–0.83; iso-butyrate, AUC: 0.75, 95% CI: 0.64–0.87), the sensitivity of 95.9% and specificity of 68.7%. A predictive model using clinical risk factors, including age, pre-pregnancy BMI, FPG, CHOL, TG, and LDL in T1, showed a moderate predictive power with an AUC of 0.77 (95% CI: 0.67–0.86), Sensitivity of 85.7%, specificity of 58.0%. After combining clinical risk factors with butyrate/iso-butyrate, the final risk prediction model can achieve an AUC of 0.89 (95% CI: 0.83–0.95) with 87.8% of sensitivity and 75.0% of specificity (Fig. 3A).

When it comes to the second trimester, the iso-butyrate showed a much better effect on GDM prediction with an AUC of 0.96 (95% CI: 0.90–1.00), sensitivity of 100%, and specificity of 95.7%. When combined with iso-butyrate in T2 with clinical risk factors, the predictive model can achieve an AUC of 0.97 (95% CI: 0.92–1.00) with 100% of sensitivity and 95.7% of specificity (Fig. 3B).

## Discussion

This nested case–control study evaluated the association of butyrate and iso-butyrate with the risk of GDM. We found that butyrate was negatively correlated with GDM risks, while iso-butyrate was positively related to GDM risks in the first half of pregnancy. Clinical risk factors combined with butyrate/iso-butyrate in T1 or iso-butyrate in T2 showed a superior predictive efficiency for GDM.

One cohort study conducted in the Mediterranean region of Northern Spain reported an average level and reference interval (2.5 percentile to 97.5 percentile) of butyrate and iso-butyrate in pregnant women with normal glycemia during the first and third trimesters [35]. As reported, the median level of butyrate and iso-butyrate in T1 were 0.73 μmol/L (0.16–1.01 μmol/L) and 0.45 μmol/L (0.32–1.67 μmol/L). The average level of butyrate in our study was lower than that in the previous research, while iso-butyrate was much higher. Dietary diversity may mediate differences in metabolite levels between studies. It has been reported that the Mediterranean diet (MD) score was associated with a higher concentration of butyrate [36]. Participants in the previous study made a MD the primary diet, which referred to a rich variety of plant foods (fruits, vegetables, potatoes, whole grains, legumes, nuts, seeds, etc.). Full contains complex insoluble dietary fiber provide sufficient substrate for butyrate production. However, Chinese eating habits are still omnivorous, and animal protein accounts for a large proportion.

Butyrate’s protective effect on plasma glucose level and insulin resistance has also been evidenced in type 2 diabetes population and animal studies [17, 18, 37–44]. However, current study results on the relationship between butyrate and GDM were inconsistent. A few previous studies showed a decreased level of butyrate and an impaired butyrate metabolism in women with GDM [23, 45, 46]. A previous study with a sample size of 30 women with GDM and 30 women without gestational complications explored potential serum metabolites biomarkers of GDM and related pathways. The results showed that 36 differential metabolites and corresponding metabolic pathways were identified in serum, including fatty acid metabolism, butyrate metabolism, bile secretion, and amino acid metabolism [46]. Study conducted in Beijing with 20 women with GDM and 40 healthy controls unveiled a lower butyrate in GDM group in the second and third trimester [23]. An experimental study investigated the effect of butyrate on insulin signaling defects in an in vitro model of GDM. Butyrate was found to reverse TNF-induced increases in IRS-1 serine phosphorylation and decreases in glucose uptake, indicating that the butyrate may be able to improve insulin sensitivity in GDM models [45]. Consistent with the above findings, our results also revealed a lower level of butyrate in women with GDM and a negative relationship between butyrate and GDM, both in T1 and T2. However, there was still at least one previous study reported that there was no significant difference in butyrate concentration between the GDM and control group [22, 47]. Pregnant healthy women without GDM (*n* = 20) and women with GDM in three different patterns (*n* = 31, 31, 22, respectively) were included in the analysis and liquid chromatography coupled with mass spectrometry (LC-MS/MS) was used for SCFA detection [47]. Small sample sizes, different population characters and diet habits, sampling time, and detection methods for SCFAs may account for different study results. The possible mechanism of butyrate in glucose and insulin resistance regulation may be related to the following aspects [11, 48] 1) appetite inhibition, leading to reduced energy intake; 2) intestinal barrier protection. It can protect the body from endotoxemia and chronic low-grade systemic inflammation; 3) free fat acid receptors (FFAR) activation or histone deacetylase inhibition. It may affect the expression of glucose regulation related genes or the secretion of glucose regulatory hormones directly or indirectly; 4) Immunosuppression. Butyrate may inhibit the expression of inflammatory factors and systemic inflammatory response; 5) β cell protection.

As part of branched short chain fatty acids (BSCFAs), iso-butyrate was mainly produced from the formation of BCAAs (including leucine, isoleucine or valine) by gut microbiota. Data on the metabolic effects of iso-butyrate is sparse. An animal study proposed for the first time claimed that protein derived from the western diet-induced insulin resistance in mice by increasing the level of iso-butyrate [49]. Results from human study also showed an increased level of iso-butyrate in the GDM group with a small sample size (*n* = 28 in the GDM group and *n* = 27 in the healthy pregnant women group) [50]. Similarly, our results revealed a positive relationship between iso-butyrate concentration and GDM risks. The superior predictive effect using iso-butyrate in the second trimester combined with clinical risk factors with an AUC of 0.97 also indicates that iso-butyrate may be a prospective biomarker for GDM diagnosis in the second trimester. However, a contrary conclusion was made through an experimental study conducted in vitro. It found that iso-butyrate has effects on adipocyte lipid and glucose metabolism that can contribute to improved insulin sensitivity in individuals with disturbed metabolism [51]. The difference in the environment of vivo and vitro may mediate the contradictory results. Limited research focused on the mechanism of iso-butyrate on GDM or insulin resistance. Only one study published in Nature Communications in 2021 proclaimed that elevated BSCFAs were associated with insulin resistance and glucose intolerance in mice [49]. The study further showed that the BSCFA, iso-butyrate and iso-valerate, induced hepatocytes insulin resistance by potentiating hepatic mTORC1/S6K1 signaling [49]. Further studies are needed to confirm the relationship between iso-butyrate and glucose regulation and clarify the possible mechanisms.

Since the different sources of fermentation substrate of butyrate and iso-butyrate, complex carbohydrates and dietary protein respectively, butyrate/iso-butyrate may reflect the balance of dietary nutrition intake to some extent and be a good health indicator. A cross-sectional study declared a tendency for butyrate levels to be higher in vegetarians (high dietary fiber intake) than omnivores, while iso-butyrate levels were lower in vegetarians [16]. A high-protein/low-fiber diet shifts the utilization of dietary to endogenously supplied proteins, causing elevated levels of cytotoxic and pro-inflammatory metabolites (such as iso-butyrate) [52]. However, there were no studies focused on the relationship between the ratio and GDM. Our results suggested that butyrate/iso-butyrate may be a protector for GDM and had a better predictive effect than butyrate or iso-butyrate alone. The superior predictive efficiency of butyrate/iso-butyrate in T1 for GDM evidenced its potential as a promising biomarker for GDM prediction.

Various studies have reported that women with GDM were characterized by a lower Firmicutes/ Bacteroidetes ratio, with a depleted abundance of butyrate-product bacteria, such as Prevotella, Coprococcus, Ruminococcaceae, Eubacterium species, Roseburia, Dialister, Lachnospiraceae, et al. [53–56]. Coprococcus has been evidenced to be positively correlated with serum SCFA levels (acetate, valerate, butyrate, et al.) in women with GDM [22]. In addition, BSCFAs abundance, the marker of BCAAs fermentation, has also been reported to be related to decreased Firmicutes and increased unknown Bacteroidetes in an artificial colon model of high-protein diets [57]. Blautia, a kind of BSCFAs (mainly including iso-butyrate, iso-valerate) production bacteria, was found in higher abundance in people with GDM [58]. Moreover, It has been reported that patients with GDM were characterized by an up-regulated metabolite of valine, which was the main substrate for iso-butyrate production [55]. Random forest analysis using fecal metabolites (5-Hydroxyindoleacetic acid and valine) also showed a high diagnostic performance with an AUC of 0.843 [55]. The findings above indicate that microbiota disturbance might contribute to GDM risks through metabolites. However, direct evidence still needs to prove the possible relationship among gut microbiota, metabolisms, and GDM in further studies.

Our study proclaimed for the first time that butyrate/iso-butyrate may be a potential biomarker for GDM early prediction in views of nutrition metabolism. It may also provide a new insight for GDM prevention in views of nutrition intake. Besides, this nested case–control study relied on a prospective cohort study conducted in Beijing; we were able to obtain the accurate information required in the survey, which minimized recall bias.

Still, several limitations should be considered when explaining our results. First, we included no information on dietary nutrient intake, and we could not evaluate the relationship between dietary carbohydrate and protein intake and circulating butyrate and iso-butyrate levels; second, the sample size was relatively small, and it might not be completely transferable to the general population. Besides, the small sample size also restricted further stratified analysis. Future studies with a larger sample size and more detailed analysis are needed to confirm this study’s results.

All in all, our findings suggested that high levels of iso-butyrate and low levels of butyrate may be associated with an increased risk of GDM. As they are produced through dietary nutrients formation by gut microbiota, further studies on the association of dietary intake and butyrate or iso-butyrate concentration in plasma may help find a novel approach to nutritional intervention for GDM. Our findings may also provide new insights into the nutrition intake and the underlying mechanisms linking them to the risk of GDM to inform diet-related preventive strategies for GDM.

### Supplementary information

Table S1

## Data Availability

Data described in the manuscript, code book, and analytic code will be made available upon request to the corresponding authors.
