# Supplementary material for: Butyrate and iso-butyrate: a new perspective on nutrition prevention of gestational diabetes mellitus
Source: Nutr Diabetes. 2024 Apr 25;14:24. doi: 10.1038/s41387-024-00276-4 (PMC11043397; doi:10.1038/s41387-024-00276-4)
Supplement: Supplementary file 1 — Table S1 [file 41387_2024_276_MOESM1_ESM.pdf]

Table S1. Association of clinical risk factors with GDM

Clinical factors	OR (95% CI)	P value
Age	1.12(1.00-1.26)	0.059
Pre-pregnancy BMI	1.27(1.07-1.50)	0.005
Education	0.95(0.53-1.70)	0.870
Economy	1.23(0.72-2.11)	0.452
Gravida	1.33(0.90-1.97)	0.157
Parity	1.21(0.55-2.69)	0.635
Smoking	2.23(0.20-25.42)	0.519
Drinking	1.40(0.35-5.58)	0.631
History of illness*	2.12(0.72-6.28)	0.174
Family history of diabetes	2.12(0.72-6.28)	0.170
GWG (until 28 weeks)	1.06(0.93-1.20)	0.370
Laboratory parameter in the first trimester		
CHOL	2.15(1.16-4.00)	0.015
TG	2.72(1.16-6.34)	0.021
HDL	0.97(0.25-3.80)	0.962
LDL	2.58(1.18-5.66)	0.018
FPG	3.63(1.17-11.28)	0.026

OR: odds ratio; CI: confidence interval; GDM: gestational diabetes mellitus; BMI: body mass index; GWG: gestational weight gain; FPG, fasting plasma glucose; CHOL, cholesterol; TG, triglyceride; HDL, high-density lipoprotein; LDL, low-density lipoprotein. p value <0.05 for statistical significance.

* history of illness including stillbirth, dead-birth, natural abortion, birth history of deformed Infants, GDM history, macrosomia, polyhydramnios history, polycystic ovarian syndrome, and vulvovaginal candidiasis.
